# The Criterion Validity of the First Year Inventory and the Quantitative-CHecklist for Autism in Toddlers: A Longitudinal Study

**DOI:** 10.3390/brainsci10100729

**Published:** 2020-10-13

**Authors:** Annalisa Levante, Serena Petrocchi, Flavia Lecciso

**Affiliations:** 1 Department of History, Society, and Human Studies, University of Salento, 73100 Lecce, Italy; flavia.lecciso@unisalento.it; 2 Lab of Applied Psychology, Department of History, Society and Human Studies, University of Salento, 73100 Lecce, Italy; serena.petrocchi@usi.ch; 3 Institute of Communication and Health, Università della Svizzera Italiana, Via Buffi 13, 6900 Lugano, Switzerland

**Keywords:** autism spectrum disorder, screening, FYI, Q-CHAT, criterion validity

## Abstract

Pediatric surveillance through screening procedures is needed to detect warning signs of risk for Autism Spectrum Disorder under 24 months of age and to promote early diagnosis and treatment. The main purpose of this study is to extend the literature regarding the psychometric properties of two screening tools, the First Year Inventory (FYI) and the Quantitative-CHecklist for Autism in Toddler (Q-CHAT), testing their criterion validity. They were administered during a three-wave approach involving the general population. At T1, 657 children were tested with the FYI and 36 of them were found to be at risk. At T2, 545 were tested with the Q-CHAT and 29 of them were found to be at risk. At T3, 12 out of the 36 children with a high score on the FYI and 11 out of the 29 children with a high score on the Q-CHAT were compared to 15 typically developing children. The criterion validity was tested considering the severity of the autistic symptoms, emotional/behavioral problems, and limited global functioning as criteria. Accuracy parameters were also calculated. Furthermore, we investigated which dimension of each questionnaire better predicted the aforementioned criterion. The results corroborated the hypotheses and confirmed the criterion validity of FYI and Q-CHAT.

## 1. Introduction

Autism Spectrum Disorder (ASD) [1] is one of the most known childhood health conditions, with early-onset before 3 years of age and symptoms including a combination of social, communicative, and behavioral impairments. Several studies [2,3,4,5,6,7,8,9,10] emphasized that early interventions, especially implemented during the opportunity window for brain plasticity, improve long-term outcomes. Early interventions increase children’s performance in several areas, such as communication abilities, adaptive functioning, facial emotion production, and prosocial behaviors [2,10,11,12,13], decrease the severity of the autistic traits [6,14,15], and offer mechanisms for parents in coping with the grief resulting from the child’s diagnosis [16].

Early intervention requires early identification of risk factors followed by early diagnosis, as recommended by the American Academy of Pediatrics [17]. Several authors [18,19,20] suggested that the accuracy of the screening procedure for autism is sufficiently reliable when the child is 18-24 months of age [20,21,22,23]; however, some recent studies [21,22,24,25] reported that children as young as 12 months of age may elicit behavioral markers for autism. Among the latter, examples of behaviors that are deficient or absent in cases of autism are joint attention, declarative pointing, communication skills, pretend play, use of gestures, and object exploration [26,27,28,29,30,31,32].

Because the percentage of children with ASD correctly identified before their first birthday is low (approximately 30%), the Center for Disease Control and Prevention (CDC) [33] recently supported the introduction of mandatory surveillance conducted by pediatricians during their well-child visits. According to CDC guidelines, reliable measures are needed to maintain an effective pediatric surveillance protocol. Emerging evidence [34] suggested that a multi-step approach with the administration of different screening tools based upon the child’s age may be the best method to detect early signs of autism during the first year of life. A multi-step approach may also reduce false positives and false negatives [34], increasing the reliability of the evaluation and better supporting early diagnosis and intervention.

Following these considerations, the present study examined the criterion validity of two age-specific screening tools, which can be administered during two key developmental moments (at 12 and at 18 months of age) for the identification of early risk factors associated with autism. In particular, the first measure examined is the First Year Inventory (FYI) [35], administered when the child is 12 months old to detect early signs of autism. The second measure is the Quantitative-CHecklist for Autism in Toddlers (Q-CHAT) [36], which is completed by parents when the child is 18 months old to detect later or regressive cases of autism. We selected these two measures with the support of several recent systematic reviews [37,38,39], which considered both the FYI and Q-CHAT as appropriate and promising measures. In the next paragraph, we provided a brief analysis of the FYI and Q-CHAT.

### Psychometric Properties and Validity of the FYI and the Q-CHAT

The First Year Inventory (FYI) has been validated in nine studies. Four of the nine studies [35,40,41,42] applied the FYI on the general population. Two studies [43,44] had a retrospective design involving a clinical sample of children with autism. Lastly, three studies [45,46,47] administered the FYI on high-risk populations of siblings of children with autism.

The questionnaire, developed by Reznick et al. [35], evaluates the child’s functioning on the socio-communicative and sensory regulatory function domains, which correspond to the two primary symptoms of autism [1]. Within the same sample recruited by Reznick et al. [35], Turner-Brown and colleagues [42] demonstrated the FYI convergent validity applying the Autism Diagnostic Observation Schedule (ADOS) [48] and the Mullen Scales of Early Learning [49]. In another study, Ben-Sasson and Carter [41] demonstrated cross-cultural validity on the Israeli general population, as compared to the American data collected by Reznick et al. [35], and confirmed its convergent validity. A recent study on an Italian sample [40] reported the cross-cultural generalizability of the FYI compared to the American and Israeli data and its internal validity. Reznick et al. [35] also tested the structural validity with an exploratory factor approach, and Levante et al. [40] gave confirmation of the two-factor structure applying a confirmatory approach.

Two retrospective studies on an American [44] and an Italian [43] sample of children with ASD confirmed the validity of the questionnaire. Specifically, Watson et al. [44] reported significant associations between the FYI, ADOS [48], Autism Diagnostic Interview-Revised (ADI-R) [50], Leiter International Performance Scale-Revised [51], Mullen Scales of Early Learning [49], and Vineland Adaptive Behavior Scale [52]. The authors demonstrated that the FYI is sensitive in differentiating among children with ASD, children with other developmental disorders, and typically developing (TD) children. The second retrospective study [43] found that the socio-communicative domain, which includes receptive and expressive communication as well as social engagement, is the most compromised in children with autism and therefore seems to be a better predictor of early signs of autism than the sensory regulatory function domain.

Finally, the FYI was applied in three studies on a high-risk population of siblings of children with autism compared to TD children. Rowberry et al. [47] compared the FYI with the revised Autism Diagnostic Observation Schedule (ADOS-2) and Mullen Scales of Early Learning [49] and found correlations with the social communication and interaction skills domains but did not find correlations with the patterns of repetitive behaviors and restricted interests. Macari et al. [46] made a comparison between groups of high- and low-risk children applying the FYI. Their results showed that unusual eye contact and gestures, expressive communication, and repetitive behaviors were the most important indicators of autism at 12 months and were more likely to be detected by parents and clinicians. Finally, the study by Lee et al. [45] administered the questionnaire to a high-risk group and a group of TD children, aiming to explore which FYI domain best differentiates between the two groups. In contrast with the results of previous studies [43,47], they showed that both FYI domains played an important role in distinguishing between high-risk and TD children.

The second screening tool applied in the current study was the Quantitative-CHecklist for Autism in Toddler (Q-CHAT). Its psychometric properties have been examined in six validation studies [36,53,54,55,56,57]. Three studies with a cross-sectional design included the general population [36,56] whereas one study involved a group of children with ASD using a prospective design [55]. Three studies with a longitudinal design explored the general population [54,57] as well as a sample of low-risk children [53].

The questionnaire was developed by Allison et al. [36] to quantify the severity of autistic traits through the assessment of joint attention, pretend play, social and expressive communication, and repetitive behaviors. The first validation study included the English general population [34] and found preliminary evidence of the internal consistency and test-retest reliability. More recently, Lecciso et al. [56] demonstrated the generalizability of the Q-CHAT on the Italian population, its confirmatory structural validity, and measurement invariance by gender. Cross-sectional evidence of the accuracy of the Q-CHAT in predicting the diagnostic outcomes was found by Ruta et al. [55] through the application of the ADOS-2 [58], Griffith Developmental Mental Scale [59], and Child Behavior Checklist 1.5-5 (CBCL) [60] on an Italian population of children with ASD. The evidence demonstrated external validity to differentiate between children with autism and TD children.

A longitudinal study [57] showed convergent validity considering the evaluation of the children’s emotional/behavioral symptoms with the CBCL [60]. The authors also demonstrated the cross-cultural validity and the explorative factorial structure on the Singaporean general population. More recently, Devescovi et al. [53] revealed significant associations between the Q-CHAT, ADOS-2 [58], and Bayley Scales of Infant and Toddler Development [61] scores among Italian low-risk children. Furthermore, the authors selected four items that appeared to be more significantly related to autism including social responses, joint attention, communication gestures, and stereotyped movement of fingers.

Given the aforementioned considerations, the FYI and Q-CHAT appear to be promising measures, as indicated in previous studies [37,38,39], though several gaps exist in the development of the two measures. Further longitudinal studies are especially needed to test their properties, as also suggested by other authors [62]. As underlined in a systematic review conducted by Petrocchi et al. [37], the FYI and Q-CHAT cannot yet be considered as the gold standard, which includes instruments with the highest reliability, of the early identification of risk of ASD. The ADOS-2 [58] and ADI-R [50] are currently used as the gold standard for diagnosing autism. The observational procedure applying the ADOS-2 allows experts to detect a child’s atypical behaviors by observing one-to-one interaction. The ADI-R is a semi-structural interview administered to the parent. They are both valid instruments to make a diagnosis of ASD, and even if they have often been applied in combination, for research practice, it is still possible to administer only one of them without losing validity [63].

Another crucial point regarding the identification of an effective pediatric surveillance procedure for autism considers a multi-step approach with the administration of different screening tools at different developmental ages may be more effective, as indicated by previous studies [34]. Among these studies on the FYI and Q-CHAT, only Devescovi et al. [53] designed a two-stage screening approach applying both the Q-CHAT and Infant-Toddler Checklist [64] to screen early pre-linguistic communication deficits. Further evidence is needed regarding measures considered to be useful in detecting early signs of autism.

The present study aimed to examine the psychometric properties of the FYI and Q-CHAT as a two-step approach for the general population with a longitudinal study. More specifically, the current study intended to expand the literature regarding the criterion validity of the two screening measures following a group of children over the course of 18 months. The following hypotheses were formulated:

**Hypothesis 1.**  *Children at risk of autism at 11–13 months show greater autistic symptoms, emotional/behavioral problems, and limited global functioning compared to non-at-risk children.*

**Hypothesis 2.**  *Children at risk of autism at 18–21 months show greater autistic symptoms, emotional/behavioral problems, and limited global functioning compared to non-at-risk children.*

**Hypothesis 3.**  *Children who have been detected to be at-risk, either at 11–13 or 18–21 months, are expected to receive a diagnosis of ASD or other developmental disorders. Accuracy parameters were also calculated.*

Our study also tested two research questions (RQ):

RQ1: Which FYI domains better predict the children’s autistic symptoms and global developmental functioning at 11–13 months, according to objective and reliable clinician evaluation?

RQ2: Which QCHAT factors better predict the children’s autistic symptoms and global developmental functioning at 18–21 months, according to objective and reliable clinician evaluation?

## 2. Materials and Methods

### 2.1. Procedure

The present paper reported results from a longitudinal three-wave study. The first wave (T1) began in February 2017 and continued until September 2017, during which the FYI was administered to children 11–13 months of age. The second wave (T2) took place from September 2017 to May 2018 when Q-CHAT was administered to children 18–21 months of age. Finally, the third wave (T3) was conducted from November 2017 to September 2018 and applied measures of autistic symptoms, emotional/behavioral problems, and global developmental functioning. The reported data leveraged an existing longitudinal sample [40,56]; however, the present data and analyses have not been published elsewhere.

**Figure 1:**
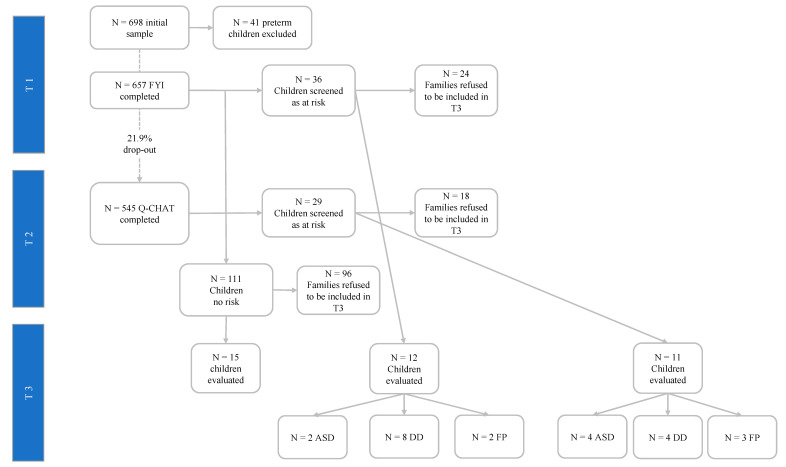
Flowchart of the longitudinal study design. ASD: Autism Spectrum Disorder; DD: developmental disorder; FP: false positive.

To recruit the participants, 115 pediatricians currently employed by the Local Public Health Service in a large city located in southern Italy were contacted via email. Sixty-four of them (55.6%) agreed to participate and received the information sheet and instructions for recruitment. All families with children born between February and September 2016 were invited to participate in T1 and T2. Figure 1 shows the flowchart of the study design of the procedure.

Three groups of children were created. The first group included children with a high-risk score on the FYI (a total score higher than the 95th percentile, corresponding to 8.15), while the second group was composed of children with a high-risk score on the Q-CHAT (a total score higher than the 95th percentile, corresponding to 43). The third group consisted of children with no risk score in either questionnaire (a total score lower than the 20th percentile in both measures). At T3, the parents of the children in all groups received a letter from their pediatrician inviting them to a free, comprehensive diagnostic evaluation for their children, administering gold-standard measures to evaluate the severity of autistic traits, emotional/behavioral problems, and global developmental functioning. Two authors of the present paper are trained developmental psychologists (A.L. & F.L.) who were blinded to the children’s risk scores and performed the psychological evaluation at T3. Children who received a diagnosis of ASD were referred to the Local Public Neuropsychiatric Health Service for the confirmative diagnosis and the intervention.

### 2.2. Measures

Socio-demographic measures of mothers, fathers, and children were collected.

#### 2.2.1. Measure of the Early Evaluation of Signs of Risk of Autism (T1/T2)

The early signs of risk of autism were measured through the FYI [65] and Q-CHAT [66].

The FYI [35] is a 63-item parent-reported screening measure assessing children’s functioning on two core symptoms of the ASD: The Social-Communication and the Sensory-Regulatory Functions domains. Three final scores were calculated, one for each domain and one for the total score (α = 0.72, ITC ≥ 0.36). Cross-cultural generalizability on the Italian population, internal validity, and retrospective concurrent validity were demonstrated elsewhere [40,43]. In the present study, the risk threshold was applied as suggested by Levante et al. [40].

The Q-CHAT is a 25-item parent-reported questionnaire assessing children’s functioning in the following areas: joint attention, social and pretend play, social interest, declarative pointing, language development, repetitive behaviors, and social communication behaviors. The questionnaire assesses three patterns of autistic traits: (1) non-social/behavioral autistic traits, (2) speech and language, and (3) joint attention/non-verbal communication. A score for each of the three patterns was calculated along with a total score as a sum of the items (α = 0.70, ITC = 0.42). Other studies demonstrated the generalizability of the Q-CHAT on the Italian population, its factorial structure, and concurrent validity [54,55,56]. In the present study, the risk threshold calculated by Lecciso et al. [56] was applied.

#### 2.2.2. Diagnostic Evaluation (T3)

To make a full diagnostic evaluation, three comparator instruments were applied: (1) ADOS-2 [58], evaluating the severity of autistic traits; (2) the Griffith Scale of Child Development (GS) [67], measuring global developmental functioning; (3) the Child Behavior Checklist 1.5–5 years (CBCL) [60], assessing emotional/behavioral problems.

The *ADOS-2* [58] is a standardized, semi-structured observational checklist evaluating children’s communication and social interaction, functional and pretend play, repetitive behaviors, and restricted interests. The ADOS-2 is applied by a trained professional and provides two partial scores—the Social Affect (SA) and Restricted and Repetitive Behaviors (RRB) dimensions—and one total score of their sum. Higher total scores indicate greater severity of autistic traits. For the present study, two trained authors (A.L. & F.L.) administered the Module Toddler of the ADOS-2 to the children involved in the diagnostic evaluation (T3).

The *GS* [67] is a standardized, structured, and child-friendly measure applied by a trained professional to assess children’s global development across five scales: (1) the foundations of learning scale (scale A), evaluating several learning abilities during toddlerhood and childhood; (2) the language and communication scale (scale B), assessing expressive and receptive language as well as social communication skills; (3) the eye and hand coordination scale (scale C), measuring fine motor skills; (4) the personal-social-emotional scale (scale D), evaluating children’s sense of self, growth, independence, social interaction, and emotional development; (5) the gross-motor scale (scale E), evaluating gross body coordination. A Global Developmental Quotient (GDQ) is calculated as the average of the raw scores of the five scales. A development quotient for all scales is also calculated according to the established norms, with higher scores indicating lower performance.

The *CBCL* [60] is a 99-item parent-reported questionnaire evaluating a child’s emotional and behavioral problems. The CBCL assesses five syndromes (affective, anxious-depressed, developmental pervasive problems, attention deficits and hyperactivity disorder, and oppositional defiant disorders) along with two clusters of internalizing (emotional reactivity, anxiety/depression, somatic complaints, and withdrawal) and externalizing problems (attention problems and aggressive behaviors). For all scales, higher scores indicate a higher risk of developing emotional and/or behavioral problems. A score for sleep problems and other unspecified problems are also calculated.

### 2.3. Participants

Six hundred fifty-seven participants completed the evaluation at T1 and according to the FYI score, 36 were found to be at risk. At T2, 545 of the 657 participants completed the Q-CHAT, with 29 receiving a risk score. Furthermore, 5 children (*n* = males) screened at-risk at T1 were screened as at risk even at T2. The other 31 (36 minus 5) identified at risk at T1 were not found to be at risk at T2. Vice versa, the other 24 (29 minus 5) at risk at T2 were not found to be at risk at T1. One hundred eleven participants received a score lower than the 20th percentile in both measures and were included in the no-risk group of children. At T3, 176 participants were invited for the diagnostic evaluation. Table 1 shows the descriptive statistics and the preliminary non-parametric comparisons among three groups invited for the diagnostic evaluation.

The criterion validity at T3 was tested on 12 out of the 36 children at risk on the FYI, 11 out of the 29 children at risk on the Q-CHAT, and 15 typical developing children. Mann–Whitney U tests were performed to compare the characteristics of families with children who completed the psychological evaluation at T3 against those who refused. Comparisons were calculated considering children’s gender and gestational age, family size, parental age, and educational level. The analyses yielded no significant results.

Comparisons among the three groups of children under evaluation at T3 were also conducted considering socio-demographic variables. Table 2 shows the results of those analyses.

### 2.4. Statistical Analysis

To compare the three groups of children on their socio-demographic variables, the Kruskal–Wallis H nonparametric one-way ANOVA was performed.

Next, the Mann–Whitney U nonparametric independent sample t-test was computed to evaluated hypotheses 1 and 2, comparing scores of gold standards measures between the groups of at-risk children and TD children. To evaluate Hypothesis 3, a confusion matrix was applied to derive sensitivity, specificity, positive predictive value, and negative predictive value. To evaluate research questions 1 and 2, the association between the diagnostic scores of the FYI domains and Q-CHAT factors were determined using Spearman’s Rho nonparametric correlations. All analyses were performed on SPSS v.25.

Finally, research questions 1 and 2 were also tested with Kendall–Theil β nonparametric regression analysis via RStudio v.1.2.5042 with the *mblm* (Median-Based Linea Models) package. The regressions considered the scores of the diagnostic instruments as the outcome and considered the FYI domains and Q-CHAT factors as predictors, respectively. Other regressions estimated the same models considering the FYI and Q-CHAT total scores.

## 3. Results

For hypothesis 1, comparisons between at-risk children on the FYI TD children on gold standard measures were performed. Table 3 shows the results of the analysis.

As hypothesized, children at risk on the FYI showed higher scores than TD children on several scales measured at T3. In particular, the findings showed that the at-risk group received higher scores on the Social Affect dimension and the total score of the ADOS-2 compared to the TD group. On the contrary, no significant difference was found between the two groups on the Restricted and Repetitive Behavior dimension. Findings showed a significant difference between the two groups in all the Griffith Scale quotients, except for the gross-motor scale. Additionally, a significant difference between the two groups on the global development quotient was found, showing that children at risk showed a lower developmental quotient than TD children. Particularly, children at risk performed most poorly on the Language and Communication and Personal/Social/Emotional scales. Finally, considering the evaluation of the children’s emotional/behavioral problems, results highlighted significant differences between the two groups on the internalizing (affective problems), externalizing (attention deficit and hyperactivity Disorders, other attention problems, and aggressive behaviors), and sleep problems. For other scales and dimensions, the group of at-risk children obtained higher scores than TD children.

For the second hypothesis, results showed significant differences between the two groups on the Social Affect dimension. The total score was evaluated by the ADOS-2, and as hypothesized, the Q-CHAT risk group received higher scores than TD children. Although the comparison was not significant, at-risk children showed a higher score on the Repetitive and Restricted Behaviors dimension compared to TD children. Findings showed significant differences between the two groups of children in all Griffith Scales, except for the gross-motor scale in the expected direction. For the risk group on the FYI, the scales with the lowest developmental quotient were Language and Communication (scale B) and Personal/Social/Emotional (scale D) scales. Lastly, results from the parental evaluation of children’s emotional and behavioral problems using the CBCL highlighted significant differences between the two groups on internalizing (affective problems) and externalizing (attention problems). On both scales, the children at risk received higher scores than TD children. Table 4 reports details of the analyses.

The diagnostic evaluation for Hypothesis 3 allowed for the clustering of the FYI risk groups into three sub-groups. The first group included children with a confirmed ASD diagnosis at T3 (n = 2 out of 12; 1 male); the ADOS-2 severity index was 4 for the female child and 8 for the male one. The second group was composed of children with diagnoses of other neurodevelopmental disorders (n = 8; 5 male), such as a communication disorder. Those children were referred to the Local Health Service for full diagnostic evaluation and intervention. The third group included two false-positive cases (2 females). There were no false-negative cases in the TD group, as all children did not fit into a diagnostic group.

Similarly, the diagnostic assessment allowed for the clustering of the Q-CHAT risk group into three sub-groups. The first group included children with a confirmed diagnosis of autism (n = 4 out of 11; 3 males); the ADOS-2 severity index ranged from 5 to 10. The second group consisted of four children with diagnoses of other neurodevelopmental disorders, such as communicable diseases. Finally, three false-positive cases were included in the third group. In the TD group, no false-negative cases were found. Sensitivity, specificity, positive predictive values, and negative predictive values were calculated through a confusion matrix, as reported in Table 5.

The examination of the first research question showed significant and positive correlations between the FYI socio-communicative domain, the SA dimension, and the total score evaluated by the ADOS-2. Furthermore, the FYI total score was significantly negative for scale D (Personal/Social/Emotional) on the Griffith Scale. Table 6 reported the *rho* and *p* values.

The regression analyses demonstrated that the FYI total score is significant and negatively predicts the development quotient of the children. Essentially, higher risk scores on the screening measure predicted delayed children’s growth, independence, social interaction, and emotional development deficits. Table 7 reported the *beta* and *p* values of the nonparametric regression models.

Similarly, to evaluate the second research question, nonparametric correlations (Table 8) and linear regression models (Table 9) were carried out. No significant association between the Q-CHAT total score and gold-standard measures was found. Results showed that Factor 1 (Non-social/Behavioral Autistic Traits) positively predicted the dimensions evaluated by the ADOS-2. Furthermore, the same factor positively predicted the Language and Communication (scale B) and Eye and Hand coordination (scale C) scores evaluated by the Griffith Scale of the children at risk. Factor 2 (Speech and Language) and Factor 3 (Joint Attention/Non-verbal Communication) showed significantly affected the children’s Social Affect symptoms evaluated by the ADOS-2. Overall, results highlighted that Factor 2 negatively predicted fine-motor (scale C) and social interaction (scale D) skills, with high-risk scores on the Q-CHAT at 18–21 months of life, predicting children’s difficulties with visual-perceptual abilities and delayed social and emotional development.

## 4. Discussion

The current study aimed to examine the psychometric properties of the FYI and Q-CHAT as a two-step approach for the general population with a longitudinal study design. The current study also aimed to expand the literature regarding the criterion validity of these two screening measures by following a group of children over the course of 18 months. The criterion validity of the two measures of risk for autism was tested through the application of instruments considered to be the gold standard for the evaluation of autism in toddlers (ADOS-2, Griffith Scale, and CBCL).

The first hypothesis proposed that children at risk on the FYI would show higher scores on the diagnostic instruments for the severity of autistic symptoms, emotional/behavioral problems as well as on the developmental quotient, as compared to TD children.

The analyses confirmed the first hypothesis. In particular, the findings showed that the children at risk received higher scores on the ADOS-2 and as determined by previous studies, when applying the FYI on general [41] and clinical populations [43,45,46,47], autistic traits more discernable at 12 months of age were related to the Social Affect dimension but unrelated to the Repetitive and Restricted Behaviors dimension. The results provided evidence that social communication impairments are present and identifiable at 12 months of age, allowing for more detection of autistic traits than behavioral patterns.

Insignificant results regarding Repetitive and Restricted Behaviors at 12 months of age could be related to the developmental trajectories of children’s playing activities. As several authors stated [68,69], between the first and the second year of life, children play repetitively with a toy for exploratory purposes and thus have restricted interests in or preferences for a particular object. However, the same authors suggested [68,69] that these specific behaviors are not prevalent and decrease with age for typically developing children.

Furthermore, Hypothesis 1 was supported by the relationship between the FYI and the children’s global functioning outcomes evaluated by the Griffith Scales [67]. Children who reached a high FYI risk score showed low performance on developmental functioning, except for gross-motor skills. This evidence is consistent with previous studies that applied the same measure [41,45,46,47] and reported a delay or deficit in learning, language, fine motor skills, and social competencies. It is worth noting that in our sample, the lowest developmental quotients concerned the Language/Communication and Personal/Social/Emotional competences. This evidence was also supported by previous studies [31,70,71] which first reported these competencies as parental concerns and compromised functioning related to autism.

Additionally, our results highlighted that high-risk scores on the FYI were related to high scores on children’s emotional/behavioral problems, as reported by parents. Specifically, our results confirmed evidence found by Iao et al. [72] who speculated that children at risk for autism at 12 months of age could also manifest comorbidities with early symptoms of internalizing, externalizing, or sleep problems, which are closely related to the autistic condition [23,73,74,75].

The second hypothesis predicted that children at risk of autism at age 18-21 months would show greater autistic symptoms, emotional/behavioral problems, and more limited global functioning compared to TD children. As predicted, the evidence corroborated this second hypothesis. First, our results showed a significant difference between the group of children with a risk score on the Q-CHAT and the group of children with no risk on the Social Affect dimension; however, results were not significant on the Repetitive and Restricted Behaviors dimension. As found to be true for the FYI, the previous literature also supports our results from the Q-CHAT [55] concerning the relationship between variables. Next, Hypothesis 2 was corroborated by the children’s developmental functioning assessment. The at-risk group showed lower performance than TD children and the FYI risk children, as evidenced in a study by Ruta et al. [55]. Third, comparisons between risk vs. no risk groups on their emotional/behavioral problems assessment confirmed findings from Ruta et al. [55] and Magiati et al. [57]. These authors found that even at 18 months of age, internalizing and externalizing problems can be present as possible comorbid conditions.

The third hypothesis stated that children detected to be at risk, either at 11–13 or 18–21 months of age, were expected to receive a diagnosis of ASD or other developmental disorders at 36 months of age. The diagnostic outcomes and results of the confusion matrices highlighted the promising nature of the measures. with the reported sensitivity values of the screening tools allowing for detection in children who later received a diagnosis of ASD. Still, the accuracy of the measures required further validation studies on general and risk populations due to the number of false-positive cases found in our sample, which negatively impacts specificity. To better understand which factor negatively influences the final score, we re-read the questionnaire to the parents and found that providing a more accurate explanation of several items led to a lower final score. Our latter suggestion may have important implications which we discuss in the following section.

To further support the criterion validity of the two measures, our study tested two research questions aimed to identify which FYI domain and Q-CHAT factor better predict diagnostic outcomes.

Regarding our first research question, our results supported the role played by the social-communicative domain of the FYI. While it did not predict the diagnostic outcomes, it was closely related to the SA dimension of the ADOS-2, as also found by previous studies [41,43,47]. Although the sensory regulatory functions domain by itself, did not predict the diagnostic outcome, the FYI total score of both domains predicted the detection of developmental impairments closely related to the autistic condition. Such conditions, as found by Ben-Sasson and Carter [41], include growth, independence, social skills, and emotional competencies evaluated by the Griffith Scales [67]. The findings support the possibility for consistent application of the FYI social-communicative domain and the total score in identifying both autistic traits and related impairments.

Our second research question highlighted the role played by each Q-CHAT factor to predict the diagnostic outcomes. All factors evaluated by the Q-CHAT were related and positively predicted the diagnostic outcomes. Moreover, Factor 2 negatively predicted fine motor and personal/social/emotional skills. Both results were consistent with the literature [76,77,78,79], which found a co-occurrence between fine motor skills and expressive language in ASD children. As suggested by some authors [80,81], a gap in one domain exists in the other. Lastly, our results further confirmed the literature [82,83,84] which underlined that the children’s language and communication skills guided social and emotional development, competencies impaired in children with ASD.

## 5. Limitations

One main limitation of the study is the sample size. Although the size was large at T1 and T2, when the sample involved children from the general population, the number of children find to be at risk to develop Autism at T3 was small. Other studies on the same field involved a similar number of participants. For example, in the last session of evaluation of their longitudinal study, Ben-Sasson and Carter [41] assessed a group of 17 children at risk. Similarly, Devescovi and colleagues [53] established a diagnostic evaluation for 14 children screened positive to the Q-CHAT. Consistently to the present one, those studies aimed to identify children without any pre-existing risk conditions, expressing early signs of risk of autism. Since autism has a prevalence of 1/87 in Italy [85], it would not expect to find a large number of children from the general population with signs of risk after a longitudinal evaluation. This is a crucial point that limits the analysis itself and the generalization of the results. For this reason, it would be essential to develop a multi-centric data collection to overcome this limitation. A second limitation is the recruitment and data collection took place in a limited geographical area, which could impact the generalizability of the results. Future studies should collect data on the general population without geographical limitations to increase the generalizability of the results.

## 6. Conclusions

Considering all findings obtained by the two measures, we propose four main considerations regarding early identification of risk factors for autism. First, the dimensions concerning social communication and interaction skills are more sensitive in detecting early signs of autism before 18 months of age. Impairments in these areas are identifiable by parents at 12 months of age with the FYI and remain stable 6 months later when the Q-CHAT is administered, and such impairments are related to the core symptoms of autism [86,87,88]. Additionally, this consideration is consistent with the principles of the social brain theory of the autistic condition, affirming that social impairment is related to the deficits in the Theory of Mind [89,90,91,92,93,94].

A second consideration demonstrates that the repetitive behaviors do not play a significant role in the early detection of autism at 12 months of age whereas, at 18 months, they are significantly related to autistic traits. These findings may suggest that it can be difficult for parents to understand what types of behaviors are considered “atypical” when the child is 12 months old; however, they are likely more able to understand what should be expected from a child at 18 months of age. This could also result from the repetitiveness and restrictiveness of behaviors becoming increasingly more severe with age and consequently more recognizable by parents.

A third consideration stems from our results supporting the need for a two-step approach [34] for early identification of the risk factors for autism. Our results supported the hypothesis that repeated assessment during regular pediatric surveillance could help in the identification of atypical developmental trajectories [53,95]. The application of two measures assure greater validity of the assessment process, especially when parents completing the questionnaires have varying levels of sensibility in detecting their child’s behaviors. This consideration leads to our fourth and final consideration. The FYI and Q-CHAT, as with many other questionnaires for early identification of autism, are affected by the quality of the relationships and trust towards the physicians [96,97], highlighting the importance of the parent-pediatrician relationship. If parents trust their pediatrician, they would likely be more willing to undergo evaluations. Moreover, as suggested by Magan-Maganto et al. [34], training for pediatricians regarding early signs of autism as well as an understanding of associated parental reactionary emotions may allow them to provide parents with a more appropriate amount of assistance and guidance throughout the evaluation process. This may lead to higher adherence to the evaluation and a lower mortality rate.

## Figures and Tables

**Table 1:** Comparison between risk groups and typically developing children.

	FYI Risk Group	X^2^(df); *p*-Value	Q-CHAT Risk Group	X^2^(df); *p*-Value	TD Group	X^2^(df); *p*-Value
Children’s Socio-Demo Variables						
N	36	/	29	/	111	/
Gender M:F	19:15	X^2^_(1)_ = −0.035; *p* = 0.85	21:8	X^2^_(1)_ = 240; *p* = 0.62	51:60	X^2^_(1)_ = 2.998 **
Age in months (mean. sd)	12.9 (2.2)		19 (2.3)		12.44 (1)	
Term pregnancy (N%)	11 (30.6)	X^2^_(1)_ = 0.063; *p* = 0.80	21 (72.4)	X^2^_(1)_ = 0.094; *p* = 0.76	79 (71.2)	X^2^_(1)_ = 219
Birth weight (mean. sd)	3.059 (0.62)		3.410 (0.54)		3.310 (0.45)	
Family size	First-born = 6; Second-born or more born = 16	X^2^_(1)_ = 0.033; *p* = 0.86	First-born = 12; Second-born or more = 12	X^2^_(1)_ = 1.510; *p* = 0.22	First-born = 46; Second-born or more = 48	X^2^_(1)_ = 0.443 *
**Parent’s Socio-Demo Variables**						
Mother’ age (mean. sd)	34.19 (6.1)		32.9 (7.4)		34.16 (5.2)	
Level of maternal education	High = 22; Low = 12	X^2^_(1)_ = 1.376; *p* = 0.24	High = 4; Low = 21	X^2^_(1)_ = 0.070; *p* = 0.79	High = 46; Low = 59	X^2^_(1)_ = 2.751 **
Father’ age (mean. sd)	38.18 (7.6)		36.82 (7.97)		37.61 (5.8)	
Level of paternal education	High = 19; Low = 15	X^2^_(1)_ = 260; *p* = 0.61	High = 3; Low = 18	X^2^_(1)_ = 0.810; *p* = 0.37	High = 32; Low = 71	X^2^_(1)_ = 5.144 *

“high” level of parental education = 18 or more years of education; “low” level of parental education = up to 13 years of education. * *p* < 0.05, ** *p* < 0.010.

**Table 2:** Comparison between risk groups and typically developing children who agreed to the psychological evaluation.

	FYI Risk Group	Q-CHAT Risk Group	TD Children	H (df); *p*-Value X^2^(df); *p*-Value
Children’s Socio-Demo Variables				
N	12	11	15	/
Gender M:F	6:6	8:3	10:5	X^2^_(29)_ = 1.405
Age in months (mean. sd)	12.5 (0.54)	19.6 (3.6)	12.11 (0.92)	H_(2)_ = 23.717 ***
Term pregnancy (N.%)	8 (66.7)	8 (72.7)	9 (60)	X^2^_(2)_ = 0.524
Birth weight (mean. sd)	2.744 (1.16)	3.113 (1.22)	3.340 (0.671)	H_(2)_ = 1.704
Family size	First-born = 2; Second-born or more = 5	First-born = 7; Second-born or more = 3	First-born = 9; Second-born or more = 6	X^2^_(2)_ = 3.033
**Parental Socio-Demo Variables**				
Mother’s age (mean. sd)	35.9 (5.3)	33.18 (6.8)	34.73 (4.33)	H(2) 1.482
Level of maternal education	High = 9; Low = 3	High = 6; Low = 3	High = 12; Low = 2	X^2^_(2)_ = 1.175
Father’s age (mean. sd)	41.64 (5.2)	39.36 (8.6)	36.87 (5.4)	H(2)= 4.142
Level of paternal education	High = 6; Low = 5	High = 4; Low = 4	High = 12; Low = 2	X^2^_(2)_ = 4.013

“high” level of parental education = 18 or more years of education; “low” level of parental education = up to 13 years of education. *** *p* < 0.001.

**Table 3:** Mean scores, standard deviation, and Mann–Whitney U tests.

	FYI Risk Children M(ds)	TD Children M(ds)	*U* Mann–Whitney
Autism Diagnostic Observation Schedule -2			
Social Affect	4 (4.02)	0.87 (1.12)	U = 24.500 ***
Restricted and Repetitive Behavior	0.58 (1.44)	0.27 (0.45)	U = 89.500
Total score	4.58 (5.4)	1.13 (1.19)	U = 32.500 **
**Griffith Scale of Child Development—Developmental Quotient**			
A—Foundation of Learning	91.17 (13.67)	102.87 (11.35)	U = 41.000 *
B—Language and Communication	66.25 (35.35)	105.93 (18.14)	U = 23.500 ***
C—Eye and Hand Coordination	92.83 (16.15)	106.8 (11.57)	U = 40.000 *
D—Personal-Social-Emotional	86.67 (30.77)	106.27 (15.83)	U = 42.500 *
E—Gross-motor	101.92 (14.91)	100.73 (10.59)	U = 89.500
GDQ	82.42 (29.73)	105.93 (13.18)	U = 33.500 *
**Children’s Behavior Checklist**			
Affective problems	3.17 (2.48) [57.58]	0.67 (1.04) [51.07]	U = 36.500 **
Anxiety problems	4.08 (3.15) [50.75]	2.33 (1.67) [51.60]	U = 60.000
Pervasive problems	3.50 (2.97) [55.75]	1.67 (1.49) [51.40]	U = 56.000
Attention Deficits and Hyperactivities	5.58 (3) [52]	2.87 (2.39) [50]	U = 43.500 *
Oppositional Defiant Disorder	2.83 (1.90) [50.92]	1.67 (1.45) [50.20]	U = 56.000
Emotionally reaction	2.67 (3.55) [49.83]	1.07 (1.22) [51]	U = 71.500
Anxiety/Depression	3.08 (3.15) [49.17]	2.07 (1.58) [51.93]	U = 79.500
Somatic complaints	2.75 (2.73) [56.83]	1.20 (1.37) [52]	U = 59.500
Withdrawn	1.58 (1.97) [54.42]	0.53 (0.83) [51]	U = 56.000
Sleep problems	5 (4.41) [59.92]	2 (2.20) [51.73]	U = 54.500 *
Attention	3.50 (2.71) [57.17]	1.13 (1.30) [50.87]	U = 37.000 **
Aggressive behaviors	10.17 (7.31) [54.42]	5.13 (4.03) [47.8]	U = 49.500 *
Internalizing problems	10.08 (9.35) [51.33]	4.87 (3.96) [43.4]	U = 54.00
Externalizing Problems	13.67 (9.33) [51.08]	6.27 (4.48) [40.93]	U = 43.500 *
Total score	16 (10.92) [40.17]	6.60 (3.91) [33.4]	U = 29.00 **

GDQ: General Developmental Quotient. * *p* < 0.05, ** *p* < 0.010, *** *p* < 0.001. Mean scores of the T-scores of the CBCL scales were reported in square brackets.

**Table 4:** Mean scores, standard deviation, and Mann–Whitney U tests.

	Q-CHAT Risk Children M(ds)	TD Children M(ds)	*U* Mann–Whitney
Autism Diagnostic Observation Schedule -2			
Social Affect	6.91 (8.4)	0.87 (1.12)	U = 41.000 **
Restricted and Repetitive Behavior	2 (2.05)	0.27 (0.46)	U = 61.000
Total score	8.91 (10.26)	1.13 (1.19)	U = 44.500 *
**Griffith Scale of Child Development—Developmental quotient**			
A—Foundation of Learning	86.64 (13.13)	102.87 (11.35)	U = 32.500 **
B—Language and Communication	52.45 (51.38)	105.93 (18.14)	U = 29.500 **
C—Eye and Hand Coordination	88.18 (16.86)	106.8 (11.57)	U = 29.000 **
D—Personal-Social-Emotional	83.73 (13.71)	106.27 (15.83)	U = 21.000 ***
E—Gross Motor	100.73 (10.59)	96.08 (10.80)	U = 74.000
GDQ	79.45 (19.37)	105.93 (13.18)	U = 23.000 ***
**Children’s Behavior Checklist**			
Affective problems	2.75 (2.73) [55.60]	0.67 (1.05) [51.07]	U = 44.500 *
Anxiety problems	3.17 (2.7) [53.1]	2.33 (1.68) [51.6]	U = 85.500
Pervasive problems	3.67 (4.27) [56.7]	1.67 (1.50) [51.4]	U = 79.000
Attention Deficits and Hyperactivities	4 (2.76) [52.3]	2.87 (2.39) [51.33]	U = 66.500
Opposition Defiant Disorder	2 (2.41) [51.1]	1.67 (1.45) [50.47]	U = 87.500
Emotionally reaction	2.75 (3.54) [53.6]	1.07 (1.22) [51.07]	U = 67.000
Anxiety/Depression	3 (3.19) [53.6]	2.07 (1.58) [52.2]	U = 79.500
Somatic complaints	2.08 (2.43) [52.8]	1.20 (1.37) [52.2]	U = 68.000
Withdrawn	2.08 (2.71) [56.4]	0.53 (0.83) [51]	U = 65.500
Sleep problems	3.08 (3.45) [53.8]	2 (2.20) [52.67]	U = 76.500
Attention	2.75 (3.45) [53.4]	1.13 (1.30) [50.87]	U = 48.500 *
Aggressive behaviors	7.50 (8.04) [51.5]	5.13 (4.03) [50.53]	U = 82.500
Internalizing problems	9.92 (10.27) [47.7]	4.87 (3.96) [43.4]	U = 71.000
Externalizing Problems	10.25 (9.93) [43.4]	6.27 (4.48) [40.93]	U = 76.500
Total score	12.83 (12.72) [36]	6.60 (3.90) [33.40]	U = 67.000

GDQ: General Developmental Quotient. * *p* < 0.05, ** *p* < 0.010, *** *p* < 0.001. Mean scores of the T-scores of the CBCL scales were reported in square brackets.

**Table 5:** Confusion matrix.

	FYI		Q-CHAT	
	Positive	Negative	Positive	Negative
**ASD**	2	2	4	3
**No ASD**	8	645	4	516

FYI = Sensitivity: 100%; Specificity: 99.69%; Positive Predictive value: 50%; Negative Predictive Values: 100%; Q-CHAT = Sensitivity: 100%; Specificity: 99.42%; Positive Predictive value: 57.14%; Negative Predictive Values: 100%.

**Table 6:** Nonparametric correlation between FYI domains and the gold standard measures.

	Sensory Regulatory Functions Domain	FYI Total Domain	ADOS-2 SA	ADOS-2 RRB	ADOS-2 Total Score	Scale D
**Socio-communicative domain**	−0.683 *	0.140	0.652 *	0.313	0.652 *	−0.122
**Sensory Regulatory Functions domain**		0.539	−0.222	0.130	−0.222	−0.455
**FYI Total Domain**			0.549	0.510	0.549	−0.745 *
**ADOS-2 SA**				0.714 *	1 **	−0.272
**ADOS-2 RRB**					0.714 *	−0.545
**ADOS-2 total score**						−0.272

SA = Social Affect; RRB = Repetitive and Restricted Behaviors; Scale D = Personal-Social-Emotional scale of Griffith. * *p* < 0.05, ** *p* < 0.010.

**Table 7:** Nonparametric regression between the FYI domain and the gold standard.

	*β*-Value	MAD	*v*-Value	*p*-Value
FYI Socio-Communicative domain				
Outcomes				
ADOS-2 Social Affect	0.32955	0.12315	37	0.0972
ADOS-2 Total score	0.32955	0.12315	37	0.0972
FYI total domain				
Outcome				
Scale D	−6.372	4.315	0	0.00195 **

MAD = median absolute deviation; Scale D = Personal-Social-Emotional scale of Griffith. ** *p* < 0.010.

**Table 8:** Nonparametric correlation between the Q-CHAT factors and gold measures.

	Factor 2	Factor 3	Q-CHAT Total Score	ADOS-2 SA	ADOS-2 RRB	ADOS-2 Total Score	Scale B	Scale C	Scale D
**Factor 1**	−0.975 **	−0.963 **	−0.556	0.892 **	0.902 **	0.892 **	−0.704 *	−0.735 *	−0.289
**Factor 2**		−963 **	0.419	0.805 *	0.820 *	0.805 *	−0.643	−0.708 *	−0.697 *
**Factor 3**			0.517	0.795 *	0.791 *	0.795 *	−0.552	−0.578	−0.527
**Q-CHAT Total score**				0.600	0.585	0.600	−0.439	−0.345	−0.289
**ADOS-2 SA**					0.982 **	1 **	−0.873 **	−0.786 *	−0.838 **
**ADOS-2 RRB**						0.982 **	−0.875 **	−0.873 **	−0.878 **
**ADOS-2 Total score**							−0.873 **	−0.786 *	−0.838 **
**Scale B**								0.846 **	0.878 **
**Scale C**									0.946 **

Factor 1 = non-social/behavioral autistic traits; Factor 2 = speech and language; Factor 3 = joint attention/non-verbal communication; SA = Social Affect; RRB = Repetitive and Restricted Behaviors; Scale B = Language and Communication; Scale C = Eye and hand Coordination; Scale D = Personal-Social-Emotional. * *p* < 0.05, ** *p* < 0.010.

**Table 9:** Nonparametric regression between Q-CHAT and gold standard measures.

	*β*-Value	MAD	*v*-Value	*p*-Value
Factor 1				
Outcomes				
ADOS-2 Social Affect	0.96944	0.04942	0	0.00781 **
ADOS-2 Repetitive and Restrictive Behavior	0.33333	0.08237	0	0.0131 *
ADOS-2 Total score	1.2722	0.1606	0	0.00781 **
Scale B	5.75	8.31	15	0.0579
Scale C	−2.1667	0.8454	35	0.0156 *
Factor 2				
Outcomes				
ADOS-2 Social Affect	1.8000	0.9884	36	0.0141 *
ADOS-2 Repetitive and Restrictive Behavior	0.8000	0.1853	28	0.0223 *
ADOS-2 Total score	2.600	1.087	36	0.0141 *
Scale C	−4.600	4.262	1	0.0207 *
Scala D	−5.60	1.52	1	0.0207 *
Factor 3				
Outcomes				
ADOS-2 Social Affect	1.5036	0.6354	36	0.00781 **
ADOS-2 Repetitive and Restrictive Behavior	0.34921	0.09884	28	0.0223 *
ADOS-2 Total score	1.837	0.638	36	0.00781 **

* *p* < 0.05, ** *p* < 0.010. MAD = median absolute deviation; Factor 1 = Non-social/behavioral autistic traits; Factor 2 = Speech and language; Factor 3 = Joint attention/non-verbal communication; Scale B = Language and communication; Scale C = Eye and hand coordination; scale D = Personal/Social/Emotional.
